# Cardiovascular and Cerebrovascular Disease Associated microRNAs Are Dysregulated in Placental Tissues Affected with Gestational Hypertension, Preeclampsia and Intrauterine Growth Restriction

**DOI:** 10.1371/journal.pone.0138383

**Published:** 2015-09-22

**Authors:** Ilona Hromadnikova, Katerina Kotlabova, Lucie Hympanova, Ladislav Krofta

**Affiliations:** 1 Department of Molecular Biology and Cell Pathology, Third Faculty of Medicine, Charles University, Prague, Czech Republic; 2 Institute for the Care of the Mother and Child, Third Faculty of Medicine, Charles University, Prague, Czech Republic; National University of Singapore, SINGAPORE

## Abstract

**Aims:**

To demonstrate that pregnancy-related complications are associated with alterations in cardiovascular and cerebrovascular microRNA expression. Gene expression of 32 microRNAs (miR-1-3p, miR-16-5p, miR-17-5p, miR-20a-5p, miR-20b-5p, miR-21-5p, miR-23a-3p, miR-24-3p, miR-26a-5p, miR-29a-3p, miR-33a-5p, miR-92a-3p, miR-100-5p, miR-103a-3p, miR-122-5p, miR-125b-5p, miR-126-3p, miR-130b-3p, miR-133a-3p, miR-143-3p, miR-145-5p, miR-146a-5p, miR-155-5p, miR-181a-5p, miR-195-5p, miR-199a-5p, miR-208a-3p, miR-210-3p, miR-221-3p, miR-342-3p, miR-499a-5p, and miR-574-3p) was assessed in placental tissues, compared between groups (35 gestational hypertension, 80 preeclampsia, 35 intrauterine growth restriction and 20 normal pregnancies) and correlated with the severity of the disease with respect to clinical signs, delivery date, and Doppler ultrasound parameters. Initially, selection and validation of endogenous controls for microRNA expression studies in placental tissues affected by pregnancy-related complications have been carried out.

**Results:**

The expression profile of microRNAs was different between pregnancy-related complications and controls. The up-regulation of miR-499a-5p was a common phenomenon shared between gestational hypertension, preeclampsia, and intrauterine growth restriction. Preeclamptic pregnancies delivering after 34 weeks of gestation and IUGR with abnormal values of flow rate in the umbilical artery demonstrated up-regulation of miR-1-3b. Preeclampsia and IUGR requiring termination of gestation before 34 weeks of gestation were associated with down-regulation of miR-26a-5p, miR-103a-3p and miR-145-5p. On the other hand, some of microRNAs (miR-16-5p, miR-100-5p, miR-122-5p, miR-125b-5p, miR-126-3p, miR-143-3p, miR-195-5p, miR-199a-5p, miR-221-3p, miR-342-3p, and miR-574-3p) were only down-regulated or showed a trend to down-regulation just in intrauterine growth restriction pregnancies requiring the delivery before 34 weeks of gestation.

**Conclusion:**

Epigenetic changes induced by pregnancy-related complications in placental tissue may cause later onset of cardiovascular and cerebrovascular diseases in offspring.

## Introduction

Preeclampsia and fetal growth restriction (FGR) are major complications affecting 2–10% of pregnancies responsible for maternal and perinatal morbidity and mortality [1, 2]. Preeclampsia usually develops after 20 weeks of gestation and is characterized by chronic or gestational hypertension combined with proteinuria [3], which results from defective placentation eliciting inadequate uteroplacental blood perfusion and ischemia [4, 5]. The causes of preeclampsia and FGR remain unknown; however, preeclampsia is thought to be an implantation disorder [6].

Hypertension in pregnancy induces long-term metabolic and vascular abnormalities that might increase the overall risk of cardiovascular, cerebrovascular, and kidney diseases, as well as diabetes mellitus, later in life [7–9]. Increasing evidence suggests an association between preeclampsia or eclampsia and the risk for latter developing hypertension, atherosclerosis, ischemic heart disease, congestive heart failure, stroke, and deep venous thrombosis, and metabolic syndrome [10–15]. Increased risk for ischemic heart disease, myocardial infarcts, heart failure, and ischemic stroke has also been observed among women with gestational hypertension [9]. Women with a history of pregnancy complicated by intrauterine growth restriction and low infant birth weight are at a higher risk for subsequent ischemic heart disease as well [16]. Epidemiologic and experimental data strongly indicate that children born to a pregnancy complicated by preeclampsia have an unique, life time cardiovascular risk profile that is present from early life, and represent a population that may benefit from early implementation of primary prevention strategies [17]. Childhood obesity, hypertension, and diabetes are the most common intermediate and long-term health consequences of fetal undernutrition caused by placental insufficiency [8, 18]. Interestingly, familial predisposition to preeclampsia occurs. Men and women exposed to preeclampsia in utero were more likely to trigger preeclampsia in their partners or develop preeclampsia, respectively [19–21].

MicroRNAs belong to the family of small noncoding RNAs (18–25 nucleotides) that regulate gene expression at the posttranscriptional level by degrading or blocking translation of target messenger RNA (mRNA) [22, 23]. MicroRNA analyses indicate that a variety of tissues display microRNA expression profiles that are significantly different from normal tissues [24], which may be useful for a wide range of applications in clinical diagnostics [25]. Recent studies have shown that preeclampsia and fetal growth restriction are associated with alterations in microRNA expression in the placenta [26–45].

The aim of the present study was to explore placental tissue expression profile of microRNAs known to be involved in the onset of diverse cardiovascular and cerebrovascular diseases (miR-1-3p, miR-16-5p, miR-17-5p, miR-20a-5p, miR-20b-5p, miR-21-5p, miR-23a-3p, miR-24-3p, miR-26a-5p, miR-29a-3p, miR-33a-5p, miR-92a-3p, miR-100-5p, miR-103a-3p, miR-122-5p, miR-125b-5p, miR-126-3p, miR-130b-3p, miR-133a-3p, miR-143-3p, miR-145-5p, miR-146a-5p, miR-155-5p, miR-181a-5p, miR-195-5p, miR-199a-5p, miR-208a-3p, miR-210-3p, miR-221-3p, miR-342-3p, miR-499a-5p, and miR-574-3p).

We focus mainly on those microRNAs playing a role in pathogenesis of dyslipidaemia (miR-1-3p, miR-21-5p, miR-33a-5p, miR-122-5p, miR-146a-5p, miR-155-5p) [46–63], hypertension (miR-21-5p, miR-143-3p, miR-145-5p, miR-181a-5p, miR-208a-3p) [64–70], vascular inflammation (miR-29a-3p, miR-126-3p, miR-146a-5p, miR-155-5p, miR-195-5p, miR-210-3p, miR-221-3p) [71–73], insulin resistance and diabetes (miR-20b-5p, miR-21-5p, miR-24-3p, miR-26a-5p, miR-29a-3p, miR-103a-3p, miR-126-3p, miR-133a-3p, miR-181a-5p) [74, 75], atherosclerosis (miR-21-5p, miR-33a-5p, miR-126-3p, miR-143-3p, miR-145-5p, miR-155-5p) [76–82], angiogenesis (miR-16-5p, miR-17-5p, miR-20a-5p, miR-21-5p, miR-92-3p, miR-100-5p, miR-126-3p, miR-210-3p, miR-221-3p) [83–85], coronary artery disease (miR-1-3p, miR-17-5p, miR-20a-5p, miR-21-5p, miR-92-3p, miR-126-3p, miR-133a-3p, miR-143-3p, miR-145-5p, miR-155-5p, miR-181a-5p, miR-195-5p, miR-208a-3p, miR-221-3p) [83, 86–91], myocardial infarction and heart failure (miR-1-3p, miR-16-5p, miR-17-5p, miR-20b-5p, miR-21-5p, miR-23a-3p, miR-24-3p, miR-26a-5p, miR-29a-3p, miR-92-3p, miR-100-5p, miR-122-5p, miR-125b-5p, miR-126-3p, miR-103a-3p, miR-133a-3p, miR-181a-5p, miR-195-5p, miR-199a-5p, miR-208a-3p, miR-210-3p, miR-499a-5p) [92–125].

To our knowledge, no study on cardiovascular and cerebrovascular microRNA expression in placental tissues derived from gestational hypertension has been carried out. Our study also describes, for the first time, placental expression of these microRNAs in preeclampsia (miR-23a-3p, miR-24-3p, miR-33a-5p, miR-92-3p, miR-100-5p, miR-103a-3p, miR-122-5p, miR-125b-5p, miR-130b-3p, miR-133a-3p, miR-143-3p, miR-145-5p, miR-146a-5p, miR-199a-5p, miR-208a-3p, miR-221-3p, miR-499a-5p, and miR-574-3p) or intrauterine growth restriction (miR-1-3p, miR-17-5p, miR-20a-5p, miR-20b-5p, miR-21-5p, miR-23a-3p, miR-24-3p, miR-26a-5p, miR-29a-3p, miR-33a-5p, miR-92-3p, miR-100-5p, miR-103a-3p, miR-122-5p, miR-125b-5p, miR-126-3p, miR-130b-3p, miR-133a-3p, miR-143-3p, miR-145-5p, miR-146a-5p, miR-155-5p, miR-181a-5p, miR-195-5p, miR-199a-5p, miR-208a-3p, miR-210-3p, miR-221-3p, miR-342-3p, miR-499a-5p, and miR-574-3p).

## Materials and Methods

### Patients

The study was retrospective. The studied cohort consisted of 170 consecutive Caucasian pregnant women involving 35 pregnancies with gestational hypertension (GH), 80 pregnancies with clinically established preeclampsia (PE), 35 pregnancies complicated by intrauterine growth restriction (IUGR), and 20 normal pregnancies. Of the 80 patients with preeclampsia, 34 had symptoms of mild preeclampsia and 46 were diagnosed with severe preeclampsia. Twenty-nine preeclamptic patients required delivery before 34 weeks of gestation and 51 patients delivered after 34 weeks of gestation. Preeclampsia occurred both in previously normotensive patients (57 cases), and was superimposed on pre-existing hypertension (23 cases). Eleven growth-retarded foetuses were delivered before 34 weeks of gestation and 24 after 34 weeks of gestation. Oligohydramnios or anhydramnios were present in 15 growth-restricted foetuses.

An examination of blood flow (Doppler ultrasonography) showed an abnormal pulsatility index (PI) in the umbilical artery (13 preeclampsia and 19 IUGR) and/or in the middle cerebral artery (11 preeclampsia and 11 IUGR). The cerebro-placental ratio (CPR), expressed as a ratio between the middle cerebral artery and the umbilical artery pulsatility indexes was below the fifth percentile in 30 cases (13 preeclampsia and 17 IUGR). Absent or reversed end-diastolic velocity waveforms in the umbilical artery occurred in 4 cases (1 preeclampsia and 3 IUGR).

The clinical characteristics of normal and complicated pregnancies are presented in Table 1.

**Table 1:** The clinical characteristics of normal and complicated pregnancies.

	Healthy pregnant women	Preeclamptic patients	IUGR patients	GH patients
	(n = 20)	(n = 80)	(n = 35)	(n = 35)
Age (years)	30 (26.5–33)	33 (30–36)	30 (27–31)	30 (28–31,5)
Blood pressure (mmHg)				
Systolic	118 (110.5–119.5)	155 (143–164.5)	120 (115–131.3)	151 (144–161,5)
Diastolic	72 (70–82)	98 (90–101)	75.5 (70–84.3)	95,5 (90,75–100)
Proteinuria (g/24h)	None	1.1 (0.58–3.59)	None	None
Gestational age at delivery (weeks)	40 (38–41)	36 (33–38)	36.5 (31–38)	39 (39–39)
Pregnancy body mass index	26.1 (24.8–27.9)	29.1 (26.4–32.0)	26.4 (24.3–28.3)	30.2 (27.6–34.8)
Fetal birth weight (grams)	3420 (3170–3750)	2650 (1650–3210)	2120 (1560–2490)	3320 (2930–3510)
Mode of delivery				
Vaginal	18 (90%)	14 (17.5%)	9 (25.7%)	25 (71.4%)
Cesarian section	2 (10%)	66 (82.5%)	26 (74.3%)	10 (28.6%)
Fetal sex				
Boy	11 (55%)	34 (42.5%)	17 (48.6%)	16 (45.7%)
Girl	9 (45%)	46 (57.5%)	18 (51.4%)	19 (54.3%)
Glukose status				
Normal	19 (95%)	77 (96.2%)	34 (97.1%)	35 (100%)
GDM/DM	1 (5%)	3 (3.8%)	1 (2.9%)	0

Data are presented as median (25–75 percentile) for continuous variables and as number (percent) for categorical variables.

Women with normal pregnancies were defined as those without medical, obstetrical, or surgical complications at the time of the study and who subsequently delivered full term, singleton healthy infants weighing > 2500 g after 37 completed weeks of gestation. Gestational hypertension was defined as high blood pressure that developed after the twentieth week of pregnancy.

Preeclampsia was defined as blood pressure > 140/90 mmHg in two determinations 4 hours apart that was associated with proteinuria > 300 mg/24 h after 20 weeks of gestation [3]. Severe preeclampsia was diagnosed by the presence of one or more of the following findings: 1) a systolic blood pressure > 160 mmHg or a diastolic blood pressure > 110 mmHg, 2) proteinuria greater than 5g of protein in a 24-hour sample, 3) very low urine output (less than 500 ml in 24 h), 4) signs of respiratory problems (pulmonary oedema or cyanosis), 5) impairment of liver function, 6) signs of central nervous system problems (severe headache, visual disturbances), 7) pain in the epigastric area or right upper quadrant, 8) thrombocytopenia, and 9) the presence of severe fetal growth restriction [3].

Fetal growth restriction was diagnosed when the estimated fetal weight (EFW), calculated using the Hadlock formula (Astraia Software GmbH), was below the tenth percentile for the evaluated gestational age, adjustments were made for the appropriate population standards of the Czech Republic. In addition to fetal weight below the threshold of the 10^th^ percentile IUGR foetuses had at least one of the following pathological finding: an abnormal pulsatility index in the umbilical artery, absent or reversed end-diastolic velocity waveforms in the umbilical artery, an abnormal pulsatility index in the middle cerebral artery, a sign of a blood flow centralisation, and a deficiency of amniotic fluid (anhydramnios and oligohydramnios).

Centralization of the fetal circulation represents a protective reaction of the fetus against hypoxia that manifests itself in redistribution of the circulation in the brain, liver and heart at the expense of the flow reduction in the periphery [126, 127]. The cerebroplacental ratio (CPR) quantifies redistribution of cardiac output by dividing Doppler indices from representative cerebral and fetoplacental vessels.

Patients with a complicated gestation demonstrating premature rupture of membranes, in utero infections, fetal anomalies or chromosomal abnormalities, and fetal demise in utero or stillbirth were excluded from the study.

All patients who participated in this study provided written informed consent. The study was approved by the Ethics Committees of the Third Faculty of Medicine, Charles University in Prague and the Institute for the Care of the Mother and Child.

### Processing of samples

Samples of placenta were collected at the Institute for the Care of the Mother and Child (Prague, CZ) and stored at −80°C until further processing.

Total RNA was extracted from 30 mg of placental tissue preserved in RNAlater (Ambion, Austin, USA) followed by an enrichment procedure for small RNAs (siRNAs, microRNAs), according to manufacturer’s instructions using a mirVana microRNA Isolation kit (Ambion, Austin, USA). To minimize DNA contamination, we treated the eluted RNA with 5 μL of DNase I (Fermentas International, Ontario, Canada) for 30 min at 37°C. Using this novel approach, a RNA fraction highly enriched in RNA species <200nt was obtained, whose concentration and quality was assessed using a NanoDrop ND-1000 spectrophotometer (NanoDrop Technologies, USA). The A(260/280) absorbance ratio of isolated RNA was 1.8–2.0, demonstrating that the RNA fraction was pure and could be used for analysis. Additionally, the A(260/230) ratio was greater than 1.6, demonstrating negligible contamination by polysaccharides.

### Reverse transcriptase reaction using a stem-loop primer

Each of the 32 microRNAs (miR-1-3p, miR-16-5p, miR-17-5p, miR-20a-5p, miR-20b-5p, miR-21-5p, miR-23a-3p, miR-24-3p, miR-26a-5p, miR-29a-3p, miR-33a-5p, miR-92a-3p, miR-100-5p, miR-103a-3p, miR-122-5p, miR-125b-5p, miR-126-3p, miR-130b-3p, miR-133a-3p, miR-143-3p, miR-145-5p, miR-146a-5p, miR-155-5p, miR-181a-5p, miR-195-5p, miR-199a-5p, miR-208a-3p, miR-210-3p, miR-221-3p, miR-342-3p, miR-499a-5p, and miR-574-3p) was reverse transcribed into complementary DNA (cDNA) using a TaqMan MicroRNA Assay, containing microRNA-specific stem-loop RT primers (Table 2), and TaqMan MicroRNA Reverse Transcription Kit (Applied Biosystems, Branchburg, USA) in a total reaction volume of 10 μL, according to manufacturer’s instructions. Reverse transcriptase reactions were performed using a 7500 Real-Time PCR system (Applied Biosystems, Branchburg, USA) with the following thermal cycling parameters: 30 minutes at 16°C, 30 minutes at 42°C, 5 minutes at 85°C, and then held at 4°C. Finally, 12 ng of the RNA template was used for each RT reaction.

**Table 2:** Characteristics of microRNAs involved in the study.

Assay name	miRBase ID	NCBI Location Chromosome	microRNA sequence
hsa-miR-1	hsa-miR-1-3p	Chr20: 61151513–61151583 [+]	5´-UGGAAUGUAAAGAAGUAUGUAU-3´
hsa-miR-16	hsa-miR-16-5p	Chr13: 50623109–50623197 [–]	5´-UAGCAGCACGUAAAUAUUGGCG- 3´
hsa-miR-17	hsa-miR-17-5p	Chr13: 92002859–92002942 [+]	5´-CAAAGUGCUUACAGUGCAGGUAG-3´
hsa-miR-20a	hsa-miR-20a-5p	Chr13: 92003319–92003389 [+]	5´-UAAAGUGCUUAUAGUGCAGGUAG-3´
hsa-miR-20b	hsa-miR-20b-5p	ChrX: 133303839–133303907 [–]	5´-CAAAGUGCUCAUAGUGCAGGUAG-3´
hsa-miR-21	hsa-miR-21-5p	Chr17: 57918627–57918698 [+]	5´-UAGCUUAUCAGACUGAUGUUGA-3´
hsa-miR-23a	hsa-miR-23a-3p	Chr19: 13947401–13947473 [–]	5´-AUCACAUUGCCAGGGAUUUCC-3´
hsa-miR-24	hsa-miR-24-3p	Chr19: 13947101–13947173 [–]	5´-UGGCUCAGUUCAGCAGGAACAG-3´
hsa-miR-26a	hsa-miR-26a-5p	Chr3: 38010895–38010971 [+]	5´-UUCAAGUAAUCCAGGAUAGGCU-3´
hsa-miR-29a	hsa-miR-29a-3p	Chr7: 130561506–130561569 [–]	5´-UAGCACCAUCUGAAAUCGGUUA-3´
hsa-miR-33a	hsa-miR-33a-5p	Chr22: 42296948–42297016 [+]	5´-GUGCAUUGUAGUUGCAUUGCA-3´
hsa-miR-92a	hsa-miR-92-3p	Chr13: 92003568–92003645 [+]	5´-UAUUGCACUUGUCCCGGCCUGU-3´
hsa-miR-100	hsa-miR-100-5p	Chr11: 122022937–122023016 [–]	5´-AACCCGUAGAUCCGAACUUGUG-3´
hsa-miR-103	hsa-miR-103a-3p	Chr20: 3898141–3898218 [+]	5´-AGCAGCAUUGUACAGGGCUAUGA-3´
hsa-miR-122	hsa-miR-122-5p	Chr18: 56118306–56118390 [+]	5´-UGGAGUGUGACAAUGGUGUUUG-3´
hsa-miR-125b	hsa-miR-125b-5p	Chr21: 17962557–17962645 [+]	5´-UCCCUGAGACCCUAACUUGUGA-3´
hsa-miR-126	hsa-miR-126-3p	Chr9: 139565054–139565138 [+]	5´-UCGUACCGUGAGUAAUAAUGCG-3´
hsa-miR-130b	hsa-miR-130b-3p	Chr22: 22007593–22007674 [+]	5´-CAGUGCAAUGAUGAAAGGGCAU-3´
hsa-miR-133a	hsa-miR-133-3p	Chr20: 61162119–61162220 [+]	5´-UUUGGUCCCCUUCAACCAGCUG-3´
hsa-miR-143	hsa-miR-143-3p	Chr5: 148808481–148808586 [+]	5´-UGAGAUGAAGCACUGUAGCUC-3´
hsa-miR-145	hsa-miR-145-5p	Chr5: 148810209–148810296 [+]	5´-GUCCAGUUUUCCCAGGAAUCCCU-3´
hsa-miR-146a	hsa-miR-146a-5p	Chr5: 159912359–159912457 [+]	5´-UGAGAACUGAAUUCCAUGGGUU-3´
hsa-miR-155	hsa-miR-155-5p	Chr21: 26946292–26946356 [+]	5´-UUAAUGCUAAUCGUGAUAGGGGU-3´
hsa-miR-181a	hsa-miR-181a-5p	Chr9: 127454721–127454830 [+]	5´-AACAUUCAACGCUGUCGGUGAGU-3´
hsa-miR-195	hsa-miR-195-5p	Chr17: 6920934–6921020 [–]	5´-UAGCAGCACAGAAAUAUUGGC-3´
hsa-miR-199a	hsa-miR-199a-5p	Chr19: 10928102–10928172 [–]	5´-CCCAGUGUUCAGACUACCUGUUC-3´
hsa-miR-208	hsa-miR-208a-3p	Chr14: 23857805–23857875 [–]	5´-AUAAGACGAGCAAAAAGCUUGU-3´
hsa-miR-210	hsa-miR-210-3p	Chr11: 568089–568198 [–]	5´-CUGUGCGUGUGACAGCGGCUGA-3´
hsa-miR-221	hsa-miR-221-3p	ChrX: 45605585–45605694 [–]	5´-AGCUACAUUGUCUGCUGGGUUUC-3´
hsa-miR-342-3p	hsa-miR-342-3p	Chr14: 100575992–100576090 [+]	5´-UCUCACACAGAAAUCGCACCCGU-3´
mmu-miR-499	hsa-miR-499a-5p	Chr20: 33578179–33578300 [+]	5´-UUAAGACUUGCAGUGAUGUUU-3´
hsa-miR-574-3p	hsa-miR-574-3p	Chr4: 38869653–38869748 [+]	5´-CACGCUCAUGCACACACCCACA-3´

### Relative quantification of microRNAs by real-time PCR

4 μL of cDNA, corresponding to each selected microRNA, were mixed with specific primers and the TaqMan MGB probe (TaqMan MicroRNA Assay, Applied Biosystems, Branchburg, USA), and the ingredients of the TaqMan Universal PCR Master Mix (Applied Biosystems, Branchburg, USA) in a total reaction volume of 20 μL. TaqMan PCR conditions were set as described in the TaqMan guidelines. The analysis was performed using a 7500 Real-Time PCR System. All PCRs were performed in duplicates. Multiple negative controls such as NTC (water instead of cDNA sample), NAC (non-transcribed RNA samples), and genomic DNA (isolated from equal biological samples) did not generate any signal during PCR reactions. Each sample was considered positive if the amplification signal occurred before the 40^th^ threshold cycle (Ct < 40).

The expression of particular microRNA was determined using the comparative Ct method [128] relative to normalization factor (geometric mean of two selected endogenous controls) [129]. Stability of candidate endogenous controls was evaluated using NormFinder (available: http://moma.dk/normfinder-software), an ANOVA-based model, which returns standard deviation (SD) value, accumulated SD (Acc.SD) value and stability value, named variability [130–132]. In total, expression of 19 candidate endogenous controls (HY3, RNU6B, RNU19, RNU24, RNU38B, RNU43, RNU44, RNU48, RNU49, RNU58A, RNU58B, RNU66, RPL21, U6 snRNA, U18, U47, U54, U75, and Z30) was investigated in placental tissue samples.

The identification and validation analyses of suitable endogenous controls for normalization in placental tissues revealed that RNU58A and U54 were equally expressed between patients with normal course of gestation, preeclampsia and IUGR. In case of gestational hypertension qPCR data were normalized to U6snRNA and RNU66. These small nucleolar and nuclear RNAs also served as positive controls for successful extraction of RNA from all samples and were used as internal controls for variations during the preparation of RNA, cDNA synthesis, and real-time PCR.

RNA fraction highly enriched for small RNA isolated from the fetal part of one randomly selected placenta derived from gestation with normal course (the part of the placenta derived from the chorionic sac that encloses the embryo, consisting of the chorionic plate and villi) was used as a reference sample for relative quantification throughout the study.

### Statistical analysis

Data normality was assessed using the Shapiro-Wilk test, which showed that our data did not follow a normal distribution. Therefore, microRNA levels were compared between groups using non-parametric tests (the Mann-Whitney U test for the comparison between two groups and the Kruskal-Wallis test for the comparison among multiple groups). Since the Bonferroni correction was used to address the problem of multiple comparisons, the significance level was established at a *p-*value of p < 0.025 for the comparison between two groups and *p-*value of p < 0.017 for the comparison among multiple groups.

Data analysis was performed and box plots were generated using Statistica software (version 9.0; StatSoft, Inc., USA). Each box encompasses the median (dark horizontal line) of log-normalized gene expression values for microRNAs of interest in cohorts; the upper and lower limits of the boxes represent the 75^th^ and 25^th^ percentiles, respectively. The upper and lower whiskers represent the maximum and minimum values that are no more than 1.5 times the span of the interquartile range (range of the values between the 25^th^ and the 75^th^ percentiles). Outliers are indicated by circles and extremes by asterisks.

### Information on microRNA-gene-Disease ontology interactions

MiRDB (available: http://mirdb.org/miRDB/) and miRTar databases (available: http://mirtar.mbc.nctu.edu.tw/human/) were used to predict targets of those microRNAs that have been found to be dysregulated in placental tissues of patients with pregnancy-related complications.

MiRDB is an online database for miRNA target prediction and functional annotations. All the targets were predicted by a bioinformatics tool, MirTarget, which was developed by analyzing thousands of miRNA-target interactions from high-throughput sequencing experiments. Common features associated with miRNA target binding have been identified and used to predict miRNA targets with machine learning methods.

MicroRNA Target prediction (miRTar) is a tool that enables biologists easily to identify the biological functions and regulatory relationships between a group of known/putative miRNAs and protein coding genes. It also provides perspective of information on the miRNA targets on alternatively spliced transcripts.

Further, miRWalk database (available: http://www.umm.uni-heidelberg.de/apps/zmf/mirwalk/) and the Validated Targets module were used to provide information on experimentally verified interaction between appropriate microRNA and specific genes on human disease ontologies such as heart disease, myocardial infarction, congestive heart failure, vascular disease, cerebral infarction, hypertension, obesity, atherosclerosis, hypercholesterolemia and diabetic angiopathy, insulin resistance and diabetes [133]. miRWalk is a comprehensive database that provides information on miRNA from human, mouse, and rat on their predicted as well as validated binding sites on their target genes. Information on miRNA-target interactions on 2,035 disease ontologies (DO), 6,727 Human Phenotype ontologies (HPO) and 4,980 OMIM disorders is available.

## Results

### Selection and validation of endogenous controls for microRNA expression studies in placental tissues affected by pregnancy-related complications

**Fig 1:**
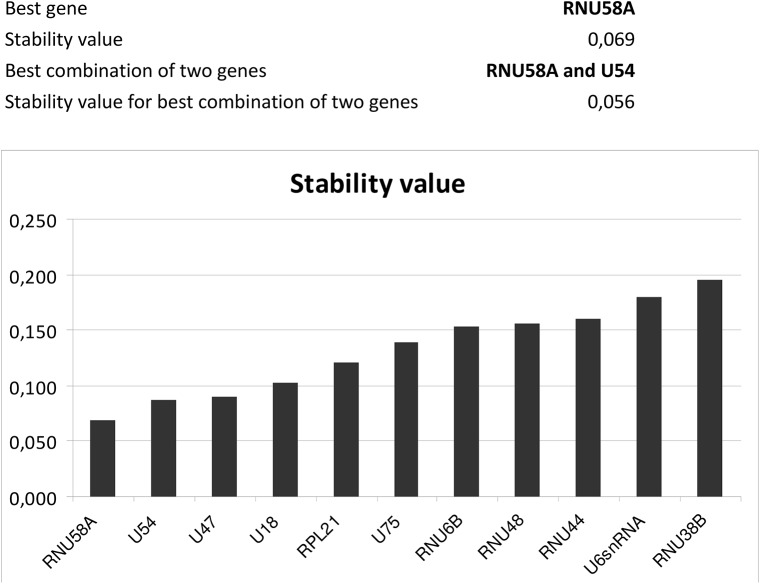
Identification of the most suitable endogenous controls in placental tissues of patients with preeclampsia and IUGR. The identification and validation analyses reveal that RNU58A and U54 are equally expressed between patients with normal course of gestation, preeclampsia and IUGR.

**Fig 2:**
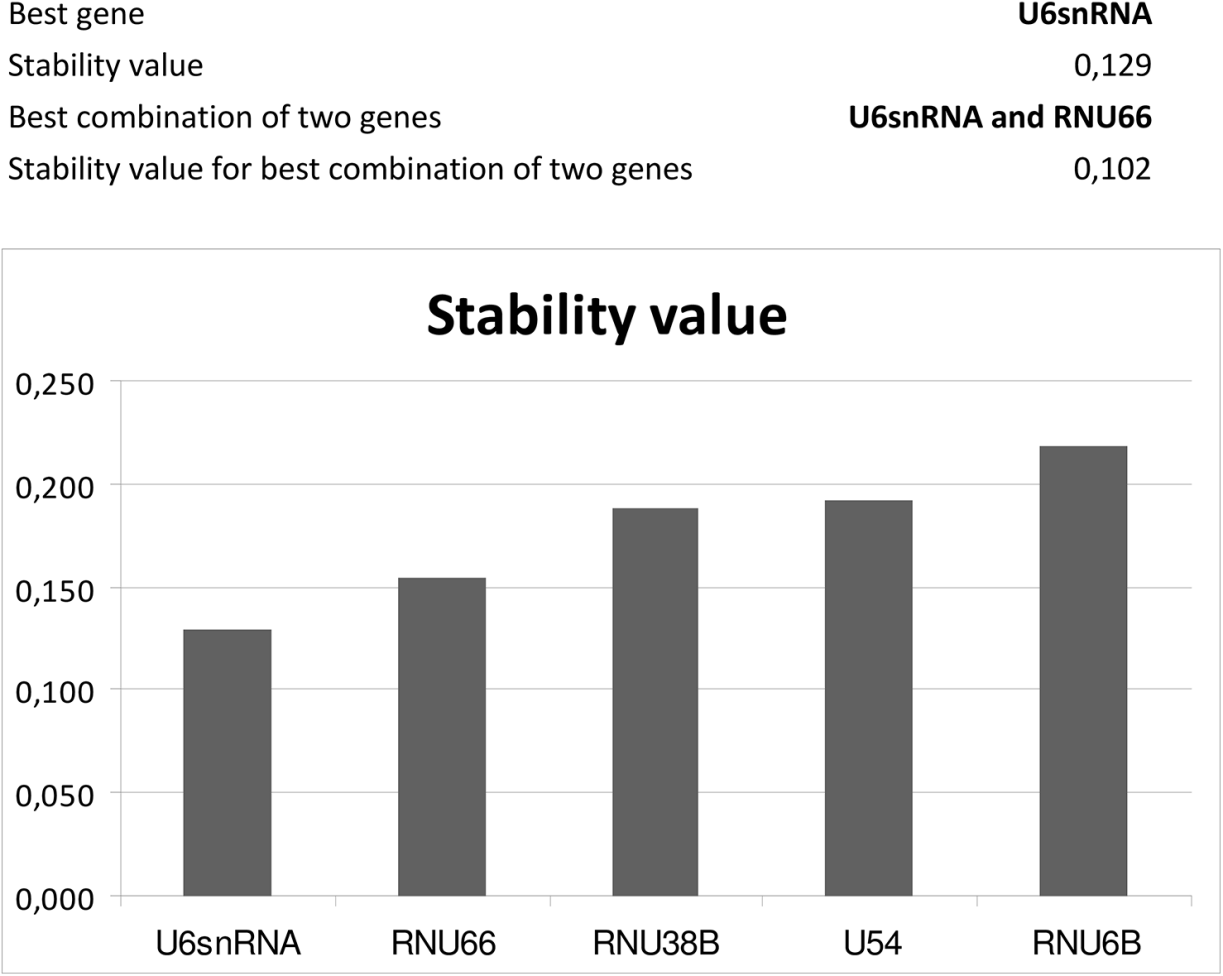
Identification of the most suitable endogenous controls in placental tissues of patients with gestational hypertension. The identification and validation analyses reveal that U6snRNA and RNU66 are equally expressed between patients with normal course of gestation and gestational hypertension.

Expression of 19 candidate endogenous controls (HY3, RNU6B, RNU19, RNU24, RNU38B, RNU43, RNU44, RNU48, RNU49, RNU58A, RNU58B, RNU66, RPL21, U6 snRNA, U18, U47, U54, U75, and Z30) was investigated in placental tissue samples obtained from 80 patients (20 normal gestation, 20 GH, 20 PE and 20 IUGR) using NormFinder [130–132]. The results were then validated using larger group of samples (20 normal gestations, 35 GH, 80 PE and 35 IUGR). In both analyses, RNU58A and U54 were identified as the most stable ncRNA and equally expressed between patients with normal and abnormal course of gestation (preeclampsia and IUGR), (Fig 1). The equivalent expression between normal gestation and gestational hypertension groups was confirmed for other two candidate endogenous controls: U6snRNA and RNU66 (Fig 2). Therefore, these small nucleolar and nuclear RNAs were selected as the most suitable endogenous controls for normalization of microRNA qPCR expression studies performed on placental tissues affected by pregnancy-related complications.

### Exclusion of miR-33a-5p and miR-208a-3p from further analyses

Unfortunately, miR-33a-5p and miR-208a-3p displayed repeatedly poor amplification curves in placental tissue samples, and therefore were excluded from further analyses.

### Cardiovascular and cerebrovascular disease associated microRNAs are dysregulated in placental tissues affected with gestational hypertension, preeclampsia and intrauterine growth restriction

Gene expression of microRNAs was compared between normal and complicated pregnancies. Gene expression of microRNAs was analysed in relation to the severity of the disease with respect to the degree of clinical signs (mild vs. severe preeclampsia, absence vs. presence of oligohydramnios or anhydramnios in growth-restricted foetuses) and delivery dates (before or after 34 weeks of gestation). Additionally, the association between microRNA gene expression and the occurrence of previous hypertension in the group of patients with preeclampsia was determined.

The association between gene expression of particular microRNAs and Doppler ultrasonography parameters (the pulsatility index in the umbilical artery, the pulsatility index in the middle cerebral artery and the cerebroplacental ratio) was analysed in the cohort of pregnancies complicated with preeclampsia or intrauterine growth restriction. Just the results that reached a statistical significance or displayed a trend toward higher or lower microRNA levels in placental tissues derived from abnormal cases are presented below.

#### Up-regulation of miR-499a-5p is a common feature of gestational hypertension, preeclampsia and IUGR

**Fig 3:**
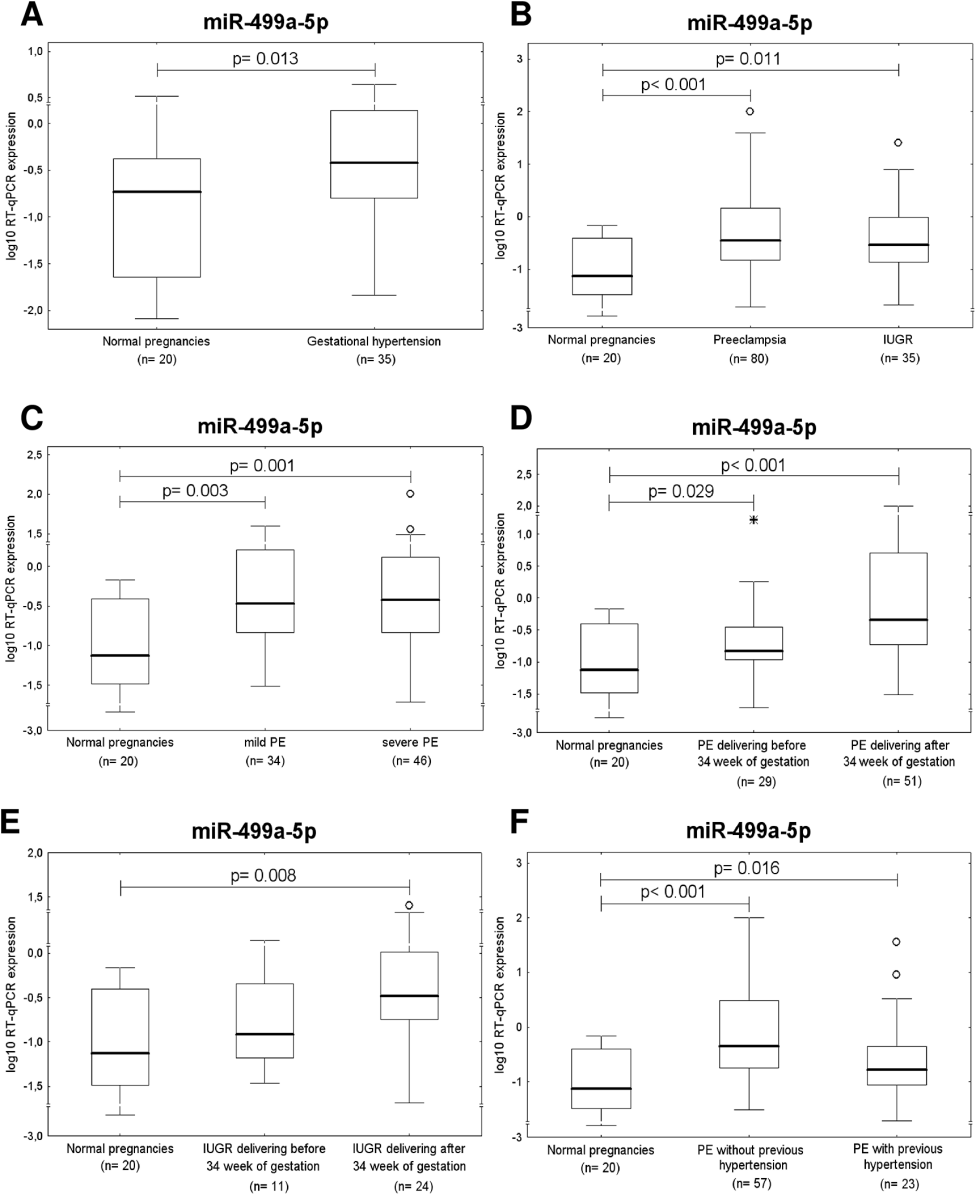
Up-regulation of miR-499a-5p is a common feature of gestational hypertension, preeclampsia and IUGR. Expression of miR-499a-5p differs significantly between the control group and pregnancies affected with (A) gestational hypertension, (B) preeclampsia and IUGR. Up-regulation of miR-499a-5p occurs in both, mild and severe preeclampsia (C). Gene expression of miR-499a-5p differs significantly between preeclamptic pregnancies delivering after 34 week of gestation and normal pregnancies (D). The difference between preeclamptic pregnancies requiring the delivery before 34 week of gestation and controls was only a trend (D). When compared to normal pregnancies, significant up-regulation of miR-499a-5p was observed in IUGR pregnancies delivering after 34 week of gestation (E). Up-regulation of miR-499a-5p appears in patients with unexpected onset of preeclampsia as well as in those with preeclampsia superposed on chronic and/or gestational hypertension (F).

After the correction for multiple comparisons, it was found that the expression of miR-499a-5p differed significantly between the control group and pregnancies affected with pregnancy complications. Higher expression rates were detected in patients with gestational hypertension (p = 0.013), preeclampsia (p< 0.001), and intrauterine growth restriction (p = 0.011), (Fig 3A and 3B).

When compared to normal pregnancies, significant up-regulation of miR-499a-5p was observed in both, mild (p = 0.003), and severe preeclampsia (p = 0.001) (Fig 3C). While gene expression of miR-499a-5p differed significantly between preeclamptic pregnancies delivering after 34 weeks of gestation and normal pregnancies (p< 0.001), the difference between preeclamptic pregnancies requiring the delivery before 34 weeks of gestation and controls was only a trend (p = 0.029) (Fig 3D). Similar results were achieved in case of IUGR (IUGR with the onset before 34 weeks of gestation: p = 0.256 and IUGR with the onset after 34 weeks of gestation: p = 0.008) (Fig 3E).

The statistical analyses revealed the difference between the group of preeclampsia superposed on chronic hypertension and/or gestational hypertension compared to control group (p = 0.016). The higher expression of *miR-499a-5p* in the group of patients with unexpected onset of preeclampsia was also observed (p< 0.001) (Fig 3F).

#### Up-regulation of miR-1-3p represents a common feature of preeclamptic pregnancies delivering after 34 week of gestation and IUGR with abnormal values of flow rate in the umbilical artery

**Fig 4:**
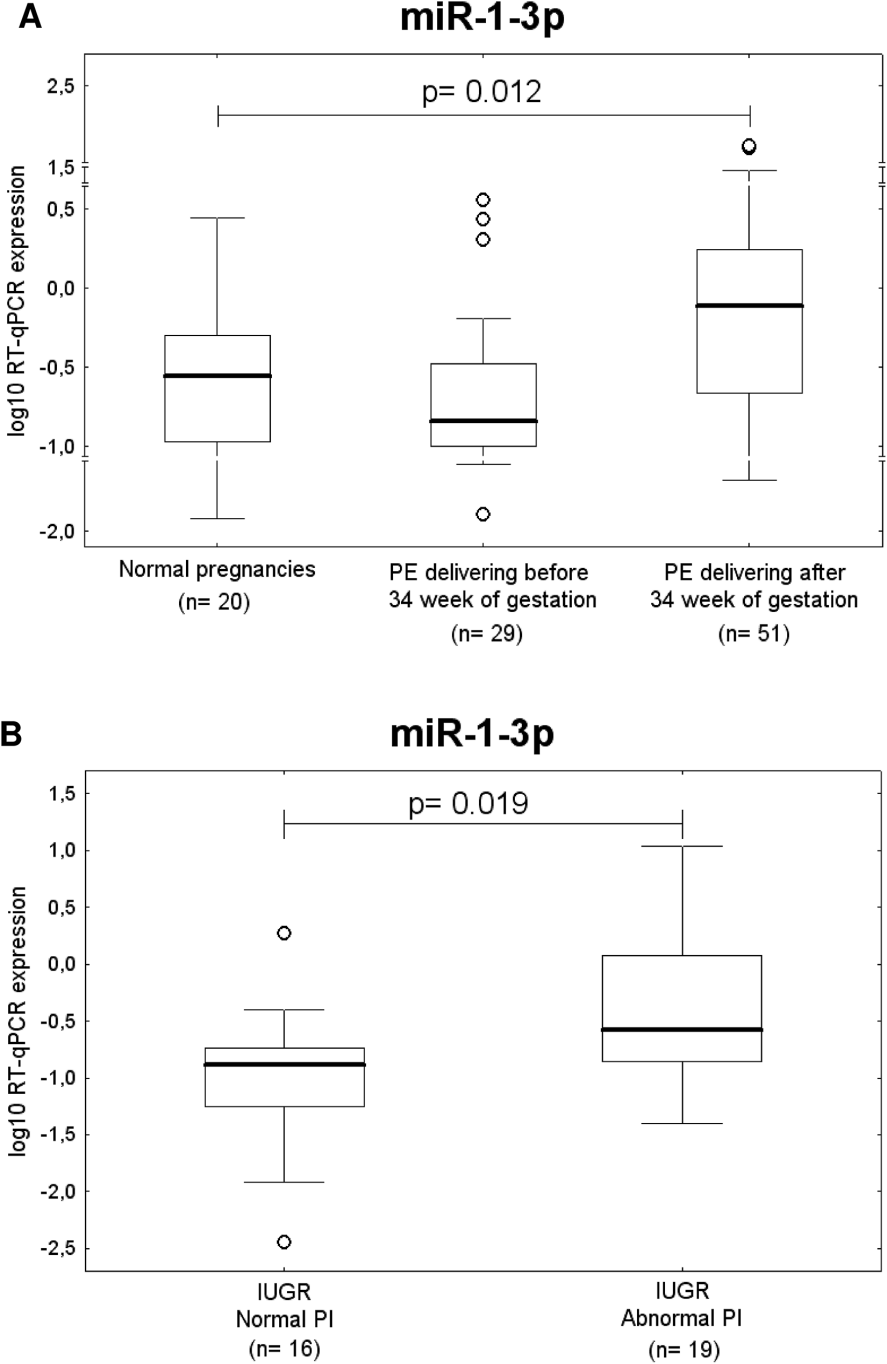
Up-regulation of miR-1-3p in pregnancy-related complications. Up-regulation of miR-1-3p represents a common feature of (A) preeclamptic pregnancies delivering after 34 week of gestation and (B) IUGR with abnormal values of flow rate in the umbilical artery. PI; pulsatility index.

After the correction for multiple comparisons, a significant difference in *miR-1-3p* expression was found between the control group and preeclampsia patients delivering after 34 weeks of gestation (p = 0.012) (Fig 4A).

IUGR pregnancies with abnormal blood flow velocity waveforms also showed significantly increased expression of *miR-1-3p* compared to IUGR patients with normal values of flow rate in the umbilical artery (p = 0.019), (Fig 4B).

#### Down-regulation of miR-26a-5p, miR-103a-3p, and miR-145-5p represents a common feature of preeclampsia and IUGR requiring the delivery before 34 weeks of gestation

**Fig 5:**
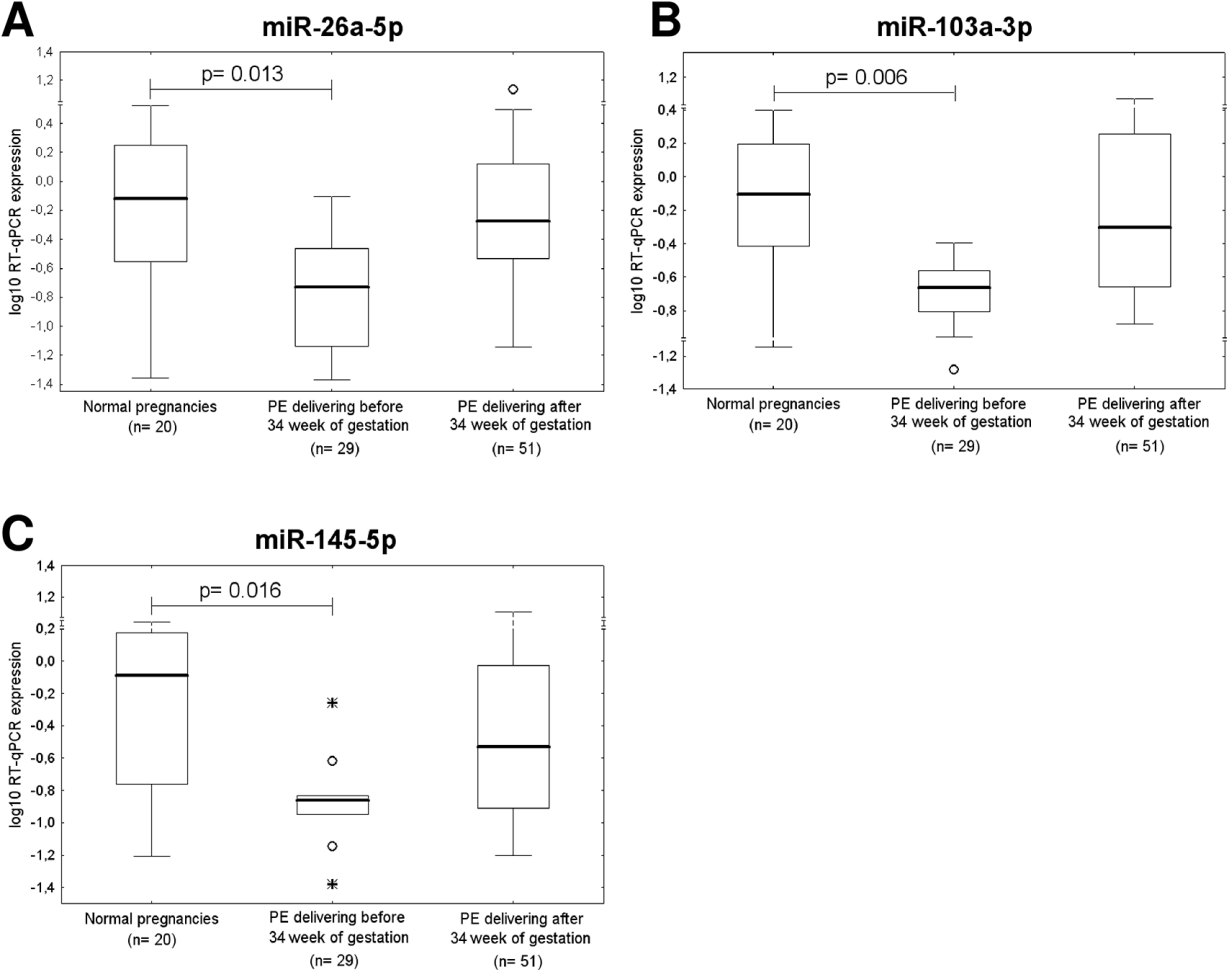
Down-regulation of miR-26a-5p, miR-103a-3p, and miR-145-5p in preeclampsia. Placental expression of (A) miR-26a-5p, (B) miR-103a-3p, and (C) miR-145-5p differs significantly between control group and pregnancies affected with preeclampsia that required delivery before 34 week of gestation.

**Fig 6:**
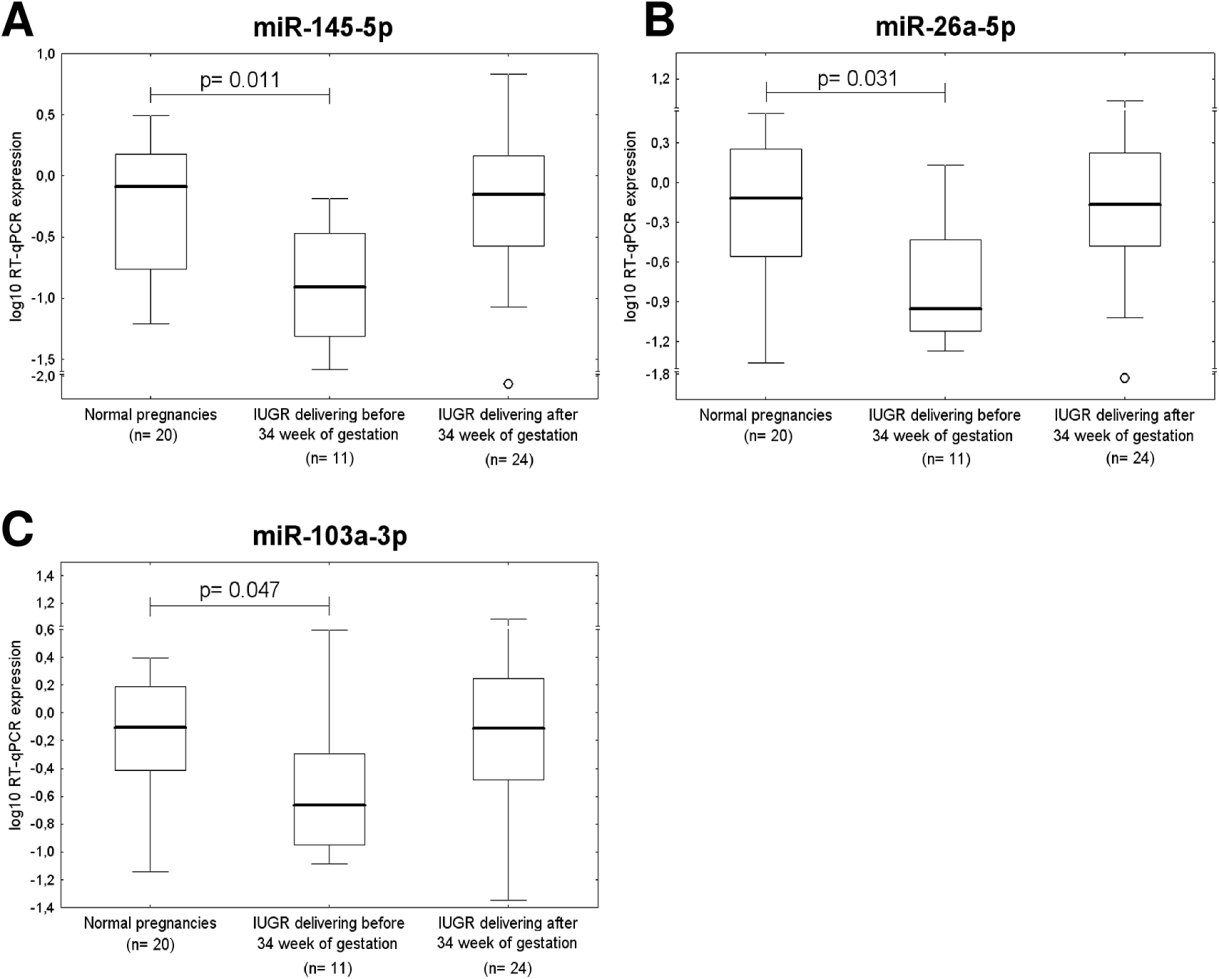
Down-regulation of miR-26a-5p, miR-103a-3p, and miR-145-5p in IUGR. Placental expression of miR-145-5p (A) differs significantly between the control group and pregnancies affected with IUGR that required delivery before 34 week of gestation. A trend toward statistical significance for down-regulation of miR-26a-5p (B) and miR-103a-3p (C) was observed for IUGR pregnancies delivering before 34 week of gestation.

After the correction for multiple comparisons, statistical analysis revealed that placental expression of *miR-26a-5p* (p = 0.013), *miR-103a-3p* (p = 0.006) and *miR-145-5p* (p = 0.016), differed significantly between the control group and pregnancies affected with preeclampsia that required delivery before 34 weeks of gestation (Fig 5A, 5B and 5C). Parallel, a significant difference in *miR-145-5p* expression (p = 0.011) was found between IUGR requiring the delivery before 34 weeks of gestation and normal pregnancy groups (Fig 6A). A trend toward statistical significance for down-regulation of *miR-26a-5p* (p = 0.031) and *miR-103a-3p* (p = 0.047) was observed for IUGR pregnancies delivering before 34 weeks of gestation (Fig 6B and 6C).

#### Down-regulation of miR-16-5p, miR-100-5p, miR-122-5p, miR-125b-5p, miR-126-3p, miR-143-3p, miR-195-5p, miR-199a-5p, miR-221-3p, miR-342-3p, and miR-574-3p represents an unique feature of IUGR requiring the delivery before 34 weeks of gestation

**Fig 7:**
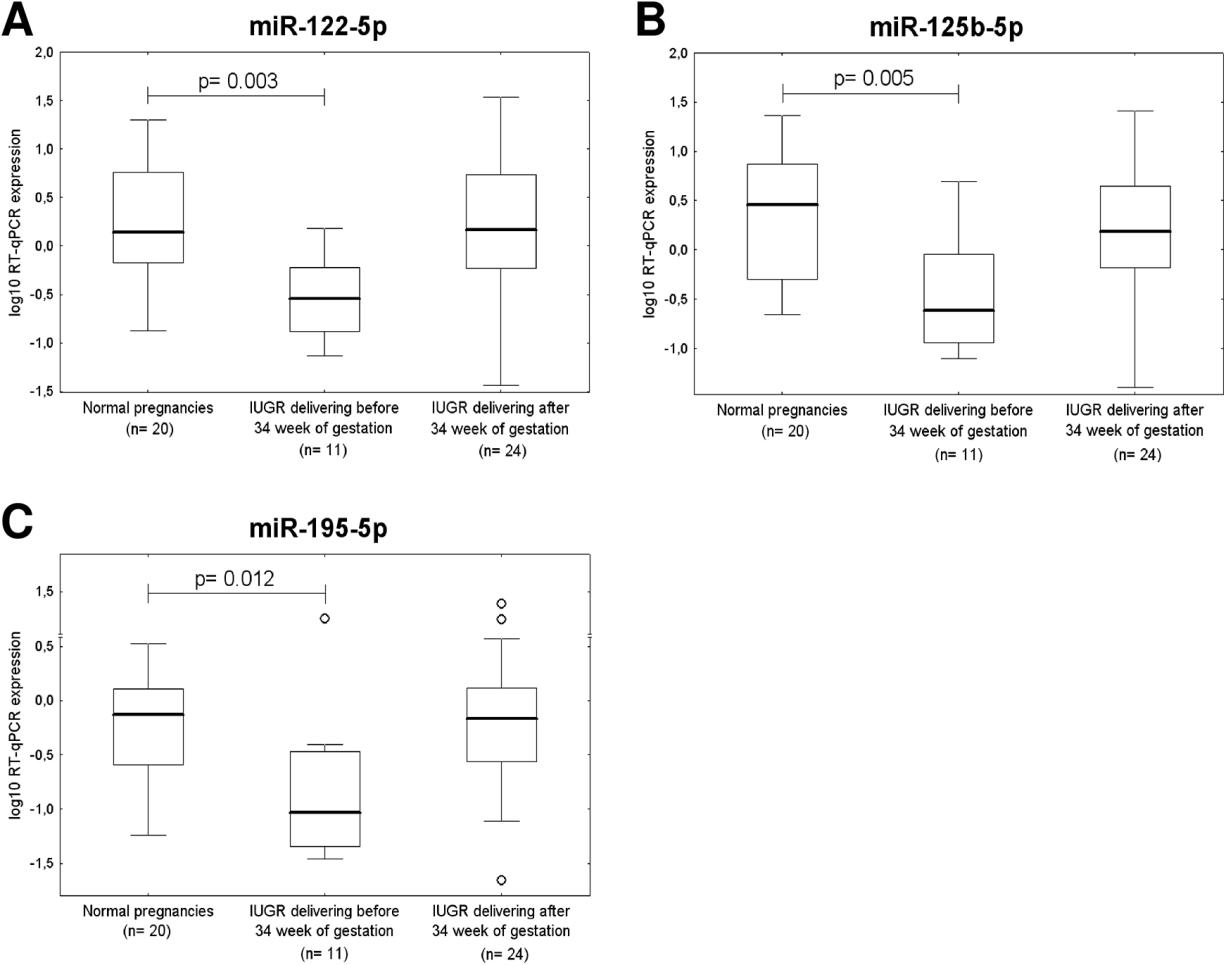
Down-regulation of miR-122-5p, miR-125b-5p, and miR-195-5p in IUGR. The expression of miR-122-5p (A), miR-125b-5p (B) and miR-195-5p (C) differs significantly between the control group and pregnancies affected with IUGR requiring the delivery before 34 week of gestation.

**Fig 8:**
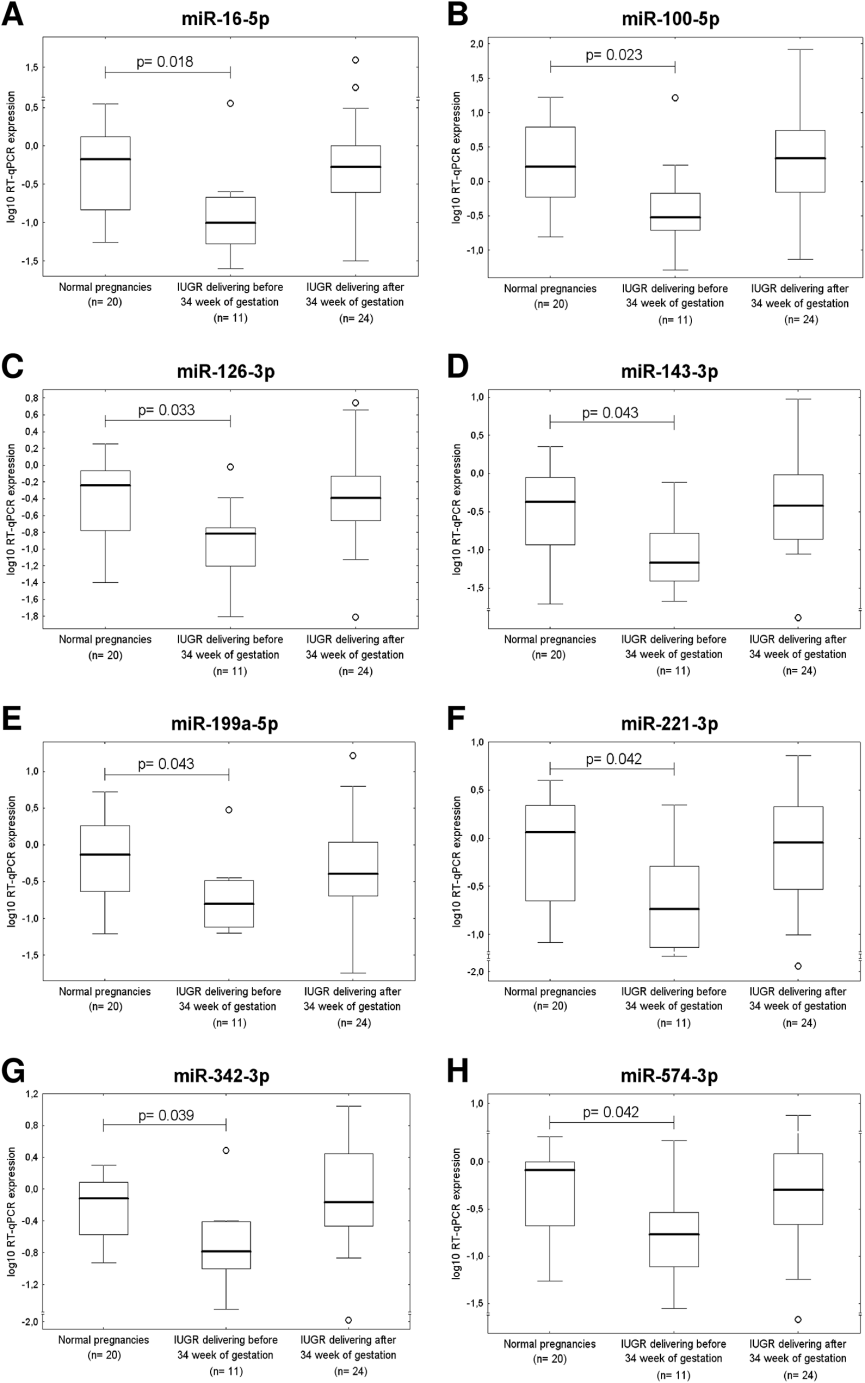
A trend to lower microRNA expression in IUGR pregnancies requiring the delivery before 34 week of gestation. MiR-16-5p (A), miR-100-5p (B), miR-126-3p (C), miR-143-3p (D), miR-199a-5p (E), miR-221-3p (F), miR-342-3p (G), and miR-574-3p (H) show a trend to down-regulation in placental tissues of IUGR pregnancies requiring the delivery before 34 week of gestation.

The down-regulation of 3 of 30 microRNAs was associated with IUGR requiring termination before 34 weeks of gestation *(miR-122-5p*, p = 0.003; *miR-125b-5p*, p = 0.005; *miR-195-5p*, *p = 0*.*012*) (Fig 7A, 7B and 7C). Several cardiovascular microRNAs showed a trend to lower expression in placental tissues derived from IUGR cases terminated before 34 weeks of gestation (*miR-16-5p*, p = 0.018; *miR-100-5p*, p = 0.023; *miR-126-3p*, p = 0.033; *miR-143-3p*, p = 0.043; *miR-199a-5p*, p = 0.043; *miR-221-3p*, p = 0.042; *miR-342-3p*, p = 0.039 and *miR-574-3p*, *p = 0*.*042)* (Fig 8A–8H).

#### Information on microRNA-gene-Disease ontology interactions

The extensive file of predicted or verified targets of all aberrantly expressed microRNAs in placental tissues derived from patients with established gestational hypertension, preeclampsia, or intrauterine growth restriction indicate that a large group of genes may be potentially dysregulated since prenatal period of life (Table 3).

**Table 3:** A list of predicted or validated targets of appropriate microRNAs dysregulated in placental tissues of patients with pregnancy-related complications in relation to cardiovascular or cerebrovascular diseases using miRWalk, miRDB and miRTar databases.

microRNA	miRWalk*	miRDB⁺	miRTar⁺	Common targets of appropriate microRNA identified in mirWalk and miRDB or miRTar databases
**miR-1-3p**	494	419	846	**69 targets:** ADAR, ALDH2, ANPEP, ARG1, BDNF, BMP7, CAPG, CAPN1, CCL2, CDK4, CEBPA, CXCL1, EDN1, EGFR, F11R, F2, FABP3, FADS1, FLNA, FN1, FOXP1, G6PD, GATA4, GCH1, GJA1, GNAI2, GSTO1, HADH, HDAC4, HMGCR, HMOX1, HSPA1A, HSPA4, HSPD1, IGF1, IL6, IL8, KCNE1, KCNN4, KIF5B, LGALS1, LIMS1, LIPC, LRP1, MEF2A, MEX3C, MFN2, NOTCH3, NR3C1, NRP1, PIM1, PROCR, PTPN1, PTPRF, SLC27A4, SP1, TCF7L2, THBS1, TIMP3, TLR4, TPM1, TRPM6, TSHR, UNC93B1, VASP, VEGFA, YWHAZ
**miR-25a-5p**	375	515	181	**38 targets**: ABCA1, ADAM9, ADM, AHR, ARHGEF1, ATP1A1, ATP1A2, BCR, CA2, CDC6, CELSR1, CFLAR, CTGF, CXADR, ESR1, FASN, FOXO3, GIT2, HSPA8, IL6, LIF, LRP6, NCEH1, NKX2-5, PALLD, PCNA, PPIA, PTER, PTGS2, RB1, SARS, SLC12A2, SMAD1, TFAM, TGFBR2, TTN, WNK1, ZNF652
**miR-103a-3p**	400	433	273	**23 targets**: APLN, ARF6, CA12, CAV1, CLOCK, CYP2C8, FGF2, FOXP1, FURIN, GPD1, ID2, INSIG1, ITGA2, KIF5B, MAP4, MAPK8, MEX3C, MTHFR, NF1, PDE4D, PIK3R1, RORA, TGFBR3
**miR-145-5p**	46	495	104	**20 targets**: ADAM17, AKR1B10, CDK4, CLOCK, CTGF, F11R, IGF1R, IRS1, IRS2, KLF5, MMP1, MMP12, MMP14, POU5F1, PPP3CA, SERPINE1, SMAD3, STAT1, VEGFA, YES1
**miR-499a-5p**	7	249	3	**0 target**
**miR-16-5p**	458	1088	1157	**89 targets**: AGER, ALDH2, AMPD1, AP2B1, APLN, APLNR, APP, ARG2, ATXN2, CA12, CACNB2, CD47, CLOCK, COL4A1, COMT, CPT1A, CUL4A, CYP27B1, EGFR, ENTPD1, F2, FASN, FDFT1, FGF2, FGFR1, FLNA, G6PD, HMOX1, HSPA1A, HSPA1B, HSPA8, HSPD1, CHUK, IFNG, IGF2R, ITGA2, JAK2, JUN, KCND3, KCNN4, KDR, KIF5B, LITAF, LRP6, MAP4, MAPK8, MERTK, MFN2, MTHFD1L, MTHFR, NAMPT, NEUROG3, NF1, NFKB1, NISCH, NRP1, PDE4D, PGLYRP1, PIK3R1, PIM1, PLAUR, POLB, PON2, PPP1R2, PRKAA1, PTGS2, RORA, RTN3, RTN4, SARS, SERPINE2, SLC12A2, SLC27A4, SLC7A1, SMAD1, SMAD5, SP1, SYT4, TFPI, TGFBR3, TIMP3, TNFRSF12A, TP53, VAMP8, VEGFA, VKORC1, WDTC1, WNT5A, XYLT1
**miR-100-5p**	371	26	229	**17 targets**: APEX1, ATP1A2, ATP2A2, BMPR2, COL4A1, DDAH1, FLT1, FOXP1, HMGB1, ID1, IGF1R, INSIG1, MMP13, MTOR, NLRP3, RB1, SMAD7
**miR-122-5p**	121	187	237	**22 targets**: ADAM10, ADAM17, ATP1A2, CDK4, FOXP1, FSTL3, GSTM3, GYS1, HLA-DQA1, HMOX1, IGF1R, KRT18, NFATC1, PHOX2A, PTPN1, RHOA, SLC7A1, SLC9A1, SOCS1, SOX2, SRF, TRIB1
**miR-125b-5p**	393	476	340	**35 targets**: ADD2, ADM, ASIC1, ATP5B, ATXN1, BMPR1B, CDKN2A, CEBPA, CYP1A1, ENPEP, ERBB3, ESRRA, HSPA1B, HSPD1, ID1, ID2, IGF2, IL1RN, IL6, ITGB3, LIF, MAPK14, MMP13, PARP1, PTGES, S100A8, SCARB2, SLC16A4, SLC7A1, STAT3, TBC1D1, TOR2A, TP53, VDR, WNK1
**miR-126-3p**	409	2	32	**10 targets**: ADAM9, CXCL12, IGFBP2, IRS1, KRAS, MERTK, PGR, SOX2, VCAM1, VEGFA
**miR-143-3p**	37	375	20	**6 targets**: AKT1, KRAS, MAPK7, MMP13, PTGS2, SERPINE1
**miR-195-5p**	374	1089	39	**36 targets**: AGER, APLN, APLNR, ATXN2, BDNF, BVES, CD47, CDK4, COMT, ENTPD1, FASN, FDFT1, FGF2, FGFR1, G6PD, HSPA1B, CHUK, JAK2, LITAF, LRP6, MAP4, MAPK9, MFN2, MTHFR, NEUROG3, NF1, PIK3R1, PPP1R2, PRKAA1, RORA, RTN3, SLC12A2, SMAD3, SYT4, VEGFA, WDTC1
**miR-199a-5p**	29	334	24	**7 targets**: CAV1, CD44, EDN1, ERBB2, HIF1A, LIF, SIRT1
**miR-221-3p**	416	316	253	**31 targets**: ADD1, ATP2A2, ATXN1, BRAP, CD4, CDKN1C, CTNNB1, CXCL12, DDAH1, ESR1, FLNA, FOXO3, ICAM1, INSIG1, ITGB3, KIT, LIMS1, LRP6, MFN2, PIK3R1, RHOA, SELE, SLC6A9, SOD2, TIMP3, TNFSF10, TP53, TRPC3, TUB, YY1, ZNF652
**miR-342-3p**	360	274	83	**16 targets**: BMP7, CXADR, GJA1, GSTA4, HNRNPC, INSIG1, ITGAM, JUN, LRP8, MAPT, NOS1AP, OSBPL8, PTPRC, PTPRN, SLC7A1, SOD2
**miR-574-3p**	358	16	2	**1 target**: RXRA

* number of verified targets in relation to human disease onthology

⁺ number of predicted targets

MiRDB and miRTar databases were used to predict targets of those microRNAs that have been found to be dysregulated in placental tissues of patients with pregnancy-related complications. miRWalk database and the Validated Targets module were used to provide information on experimentally verified interaction between appropriate microRNA and specific genes on human disease ontologies such as heart disease, myocardial infarction, congestive heart failure, vascular disease, cerebral infarction, hypertension, obesity, atherosclerosis, hypercholesterolemia, diabetic angiopathy, insulin resistance and diabetes.

## Discussion

Relative quantification of microRNA expression requires proper normalization strategy to minimize systematic and technical bias introduced at each step of microRNA quantification process [132, 134, 135]. A proper normalization of microRNA quantification requires a careful choice and validation of endogenous controls in the representative sample of the studied population [132, 136]. No previous report described an experimental identification and validation of suitable endogenous controls for normalization in pregnancy-related complications. Thus we aimed to experimentally identify the most stable endogenous controls for normalization of microRNA qPCR expression studies in gestational hypertension, preeclampsia and intrauterine growth restriction, which comprise the most common pregnancy-related complications. Analyses performed by our group revealed that RNU58A and U54 were the most stable endogenous controls in preeclamptic and IUGR placenta tissues. On the other hand, NormFinder indicated that other two non-coding small RNA (U6snRNA and RNU66) were optimal for qPCR data normalization in GH placental tissues.

Most studies on microRNA profiling in placental tissues derived from pregnancy-related complication relied on arbitrarily chosen endogenous controls. U6snRNA that is the most commonly used endogenous control, was characterized by high inter-group variation in that study when its placental tissue expression was compared between preeclampsia, IUGR and normal pregnancies.

Gene expression of cardiovascular and cerebrovascular microRNAs was compared between normal and complicated pregnancies. We focus mainly on those microRNAs being previously reported to play a role in pathogenesis of dyslipidaemia, hypertension, obesity, vascular inflammation, insulin resistance and diabetes, atherosclerosis, angiogenesis, coronary artery disease, myocardial infarction and heart failure.

Overall, the expression profile of studied microRNAs was different between complicated pregnancies and controls. With regard to individual pregnancy-related disorder subtypes, up-regulation of only 1 out of 30 studied microRNAs was found in placental tissues derived from patients with gestational hypertension, clinically established preeclampsia and intrauterine growth restriction (miR-499a-5p).

Based on the results of our study, we further studied the association between microRNA expression in placental tissues and the severity of the disease with respect to the degree of clinical signs, delivery date (before or after 34 weeks of gestation) and Doppler ultrasound examination. Cardiovascular and cerebrovascular disease associated microRNA gene expression appeared linked to the sudden onset of severe clinical symptoms requiring urgent termination of pregnancy by Caesarean section, to avoid potentially serious maternal and perinatal outcomes. Our results showed that 3 microRNAs *(miR-26a-5p*, *miR-103a-5p*, *miR-145-5p*) were dysregulated in preeclampsia requiring termination before 34 weeks and 14 microRNAs *(miR-16-5p*, *miR-26a-5p*, *miR-100-5p*, *miR-103a-5p*, *miR-122-5p*, *miR-125b-5p*, *miR-126-3p*, *miR-143-3p*, *miR-145-5p*, *miR-195-5p*, *miR-199a-5p*, *miR-221-3p*, *miR-342-3p*, and *miR-574-3p*) were altered in IUGR terminated before 34 weeks of gestation. This data suggests the involvement of these microRNAs in the pathogenesis of preeclampsia and IUGR.

Pregnancy-related complications such as gestational hypertension, preeclampsia and intrauterine growth restriction were observed to be associated with the same microRNA expression profile concerning mir-499a-5p. Nevertheless, the longer the pregnancy-related disorder lasted, the more extensive up-regulation of mir-499a-5p appeared. This suggests that the dysregulation of mir-499a-5p firstly appears during the onset of clinical symptoms of the disease, but could be intensified as a result of a sustainable development of compensatory mechanism when the disease persists for several weeks.

On the other hand, the dysregulation of miR-1-3p, which appears in preeclamptic pregnancies delivering after 34 weeks of gestation only, does not apparently drive the pathological process itself, but could be reflective of a long-term compensatory mechanism.

Limited data comparing mir-100-5p, miR-103a-3p, mir-122-5p, miR-125b-5p, miR-143-3p, miR-145-5p, miR-199a-5p, miR-221-3p, mir-342-3p, mir-499a-5p, and mir-574-3p levels between the groups of normal and complicated pregnancies are available.

With regard to miR-1-3p, our data are inconsistent with the studies of Zhu et al. [28] and Enquobahrie et al. [29] who found miR-1-3p to be significantly down-regulated in preeclamptic placentas.

The difference in the expression levels of microRNAs might be explained by different experimental approaches. Zhu et al. [28] performed the study mainly using tissue from the decidual side of the placenta (a collection of pooled tissue fragments derived from 10 randomly selected sites on the placenta) using microarray and real-time RT-PCR, with the experimental data normalised to U6snRNA. Enquobahrie et al. [29] studied homogenates of placental tissues obtained from 16 various sites by a microarray miRNA profiling followed by validation analysis using real-time PCR with normalization to miR-525-5p. While, our group focused on the analysis of microRNA gene expression in the area of the central cotyledon and experimental real-time qRT-PCR data were normalized to RNU58A and U54, experimentally identified most stable endogenous controls in preeclamptic and IUGR placenta tissues.

Our study produced similar findings to Choi et al. [38], in which they reported no significant change in miR-26a-5p levels in severe preeclamptic pregnancies delivering after 34 weeks of gestation. Nevertheless, there is no study on preeclamptic and/or IUGR pregnancies delivering before 34 weeks of gestation.

Our data also confirmed data of Maccani et al. [30] who observed as well the difference in miR-16-5p placental expression between patients with fetal growth restricted foetuses (small for gestational age) and pregnancies with normal course of gestation. Maccani et al. [30] applied real-time RT-PCR analysis on a homogenized sample derived from 12 biopsies per placenta with normalization to RNU44. While Hu et al. [27] revealed up-regulation of miR-16-5p in severe preeclamptic placentas; our study indicated no statistical significance in miR-16-5p gene expression levels. Hu et al. [27] combined large-scale profiling of microRNA expression by microarray analysis with comprehensive quantitative analysis of miRNA expression by real-time RT-PCR relative to U6snRNA. The analyses were done on frozen chorionic tissue blocks from the central part of the placenta.

Diverse studies focused on placental expression of cardiovascular and cerebrovascular microRNAs in pregnancy-related complications brought dissimilar results. Hu et al. [27] and Yang et al. [45] reported significantly increased levels of mir-126-3p and miR-195-5p in preeclamptic placentas, however Bai et al. [33], Yan et al. [39], Hong et al. [40] and Xu et al. [44] identified miR-126-3p and miR-195-5p significantly downregulated in preeclamptic placentas. Our study indicated no statistical significance in mir-126-3p and miR-195-5p gene expression levels in placental tissues affected with preeclampsia, but in pregnancies complicated by IUGR that required the delivery before 34 weeks of gestation down-regulation of mir-126-3p and miR-195-5p was observed. Similarly, multiple studies [26–29, 34–36, 42, 44] demonstrated the up-regulation of miR-210-3p, miR-181a-5p, miR-21-5p and miR-17-5p or down-regulation of miR-210-3p, miR-181a-5p, miR-21-5p and miR-17-5p in preeclamptic placentas [31, 38, 44], which was not unfortunately observed in our independent study.

Several other microRNAs such as miR-155-5p, miR-20a-5p, miR-20b-5p, and miR-29a-3p were found up-regulated in placentas from preeclampsia compared to healthy term deliveries [26, 34, 36, 41, 43] which is inconsistent with our finding.

It is important to note that only few similarities were observed. The discrepancies in microRNA placental expression may be attributed apart from no uniform way of data normalization to variability in other factors including patients´ individual characteristics (mainly race and smoking), the sampling site within the placenta, sample handling and processing. Last but not least any finding needs to be validated in large-scale studies involving sufficient number of patients within particular studied groups. Proper normalization is a critical but often underappreciated aspect of quantitative gene expression analysis. The accuracy of microarrays and quantitative RT-PCR methods, most frequently used technologies for gene expression profiling, however, is critically dependent on proper normalization of the data in as much as inappropriate normalization of qRT-PCR data can lead to incorrect conclusions [137, 138, 139].

Moreover, the power of the study for particular cases and 2 confidence levels (80% and 90%) together with minimal calculated number of subjects involved in the study should be calculated. The conclusion that expression levels of microRNAs in placental tissue differentiated between healthy pregnant women and pregnancy-related complications should not be done until the base of this statistical analysis. Determining the optimal sample size for a study assures an adequate power to detect statistical significance. Hence, it is a critical step in the design of a planned research protocol. Using too many participants in a study is expensive and exposes more number of subjects to procedure. Similarly, if study is underpowered, it will be statistically inconclusive and may make the whole protocol a failure [140].

## Conclusions

In conclusion, epigenetic changes induced by pregnancy-related complications in placental tissue may cause later onset of cardiovascular and cerebrovascular diseases in offspring.

Placental functions influence placenta itself, as well as the mother and the developing fetus, establishing a likely role in developmental programming of the fetus [141]. Alterations in blood vessel formation during placental development has the potential to reduce blood flow, leading to reduced nutrient delivery and an environment of fetal undernutrition. Fetal nutrition, or the supply of metabolic substrates delivered for growth and development, contributes to immediate, intermediate, and long-term health [142]. Immediate health consequences include intrauterine growth restriction and low birthweight. Childhood obesity, hypertension, and diabetes are the most common intermediate and long-term health consequences of fetal undernutrition [143]. Some authors postulate that early fetal and infant environment is strongly predictive for the risk of cardiovascular and cerebrovascular diseases later in life [143–147].

The main pathways by which preeclampsia or pregnancy-related complications serve to modify vascular risk would appear to be hypoxia, antiangiogenesis, endothelial dysfunction and immune modifications. These pathways individually, synergistically, or cumulatively appear to alter the epigenetic potential of placenta itself including microRNA expression profile. And so the exposition to a preeclampsia or pregnancy-related complication environment in utero leads to altered phenotype after birth [147].
